# Exchanges in a Virtual Environment for Diabetes Self-Management Education and Support: Social Network Analysis

**DOI:** 10.2196/21611

**Published:** 2021-01-25

**Authors:** Carlos A Pérez-Aldana, Allison A Lewinski, Constance M Johnson, Allison A Vorderstrasse, Sahiti Myneni

**Affiliations:** 1 School of Biomedical Informatics The University of Texas Health Science Center at Houston Houston, TX United States; 2 Durham Veterans Affairs Medical Center Durham Center of Innovation to Accelerate Discovery and Practice Transformation Durham, NC United States; 3 Duke University School of Nursing Durham, NC United States; 4 Cizik School of Nursing The University of Texas Health Science Center at Houston Houston, TX United States; 5 Rory Meyers School of Nursing New York University New York, NY United States

**Keywords:** type 2 diabetes, diabetes education, self-management, social support, virtual environments, social network analysis

## Abstract

**Background:**

Diabetes remains a major health problem in the United States, affecting an estimated 10.5% of the population. Diabetes self-management interventions improve diabetes knowledge, self-management behaviors, and clinical outcomes. Widespread internet connectivity facilitates the use of eHealth interventions, which positively impacts knowledge, social support, and clinical and behavioral outcomes. In particular, diabetes interventions based on virtual environments have the potential to improve diabetes self-efficacy and support, while being highly feasible and usable. However, little is known about the patterns of social interactions and support taking place within type 2 diabetes–specific virtual communities.

**Objective:**

The objective of this study was to examine social support exchanges from a type 2 diabetes self-management education and support intervention that was delivered via a virtual environment.

**Methods:**

Data comprised virtual environment–mediated synchronous interactions among participants and between participants and providers from an intervention for type 2 diabetes self-management education and support. Network data derived from such social interactions were used to create networks to analyze patterns of social support exchange with the lens of social network analysis. Additionally, network correlations were used to explore associations between social support networks.

**Results:**

The findings revealed structural differences between support networks, as well as key network characteristics of supportive interactions facilitated by the intervention. Emotional and appraisal support networks are the larger, most centralized, and most active networks, suggesting that virtual communities can be good sources for these types of support. In addition, appraisal and instrumental support networks are more connected, suggesting that members of virtual communities are more likely to engage in larger group interactions where these types of support can be exchanged. Lastly, network correlations suggest that participants who exchange emotional support are likely to exchange appraisal or instrumental support, and participants who exchange appraisal support are likely to exchange instrumental support.

**Conclusions:**

Social interaction patterns from disease-specific virtual environments can be studied using a social network analysis approach to better understand the exchange of social support. Network data can provide valuable insights into the design of novel and effective eHealth interventions given the unique opportunity virtual environments have facilitating realistic environments that are effective and sustainable, where social interactions can be leveraged to achieve diverse health goals.

## Introduction

### Overview

Diabetes remains a major health problem in the United States, affecting an estimated 34.2 million people of all ages (about 10.5% of the country’s population) [1]. Data show that type 2 diabetes (T2D) accounts for the most diabetes burden (between 90% and 95%), and its prevalence will continue to increase [1,2]. Diabetes is a challenging chronic illness because self-management is critical to reduce and delay the onset of complications and mortality [3-6]. Several evidence-based strategies, such as diabetes self-management education (DSME) and ongoing self-management support by peers and providers, have been shown to be effective in the management of T2D [7-9]. In particular, self-management is important in T2D given that patients manage 99% of their own care [10,11]. Moreover, diabetes self-management interventions improve diabetes knowledge and self-management behaviors, in addition to clinical outcomes [12]. Despite these benefits, less than 60% of people with diabetes attend DSME and only about 7% of newly diagnosed patients with diabetes attend DSME within 12 months following their diagnosis [13-16], indicating a pressing need for the delivery of accessible DSME and ongoing self-management support interventions.

Widespread internet connectivity provides new opportunities for wider web technology access and use by patients. Internet-based interventions, also known as eHealth, can connect patients to both peers and providers to facilitate support as well as access to evidence-based information [17]. Research suggests that T2D interventions incorporating interactive, individualized, and frequent interactions among patients, educators, and providers are among the most effective approaches [9]. eHealth interventions can provide such interactions in an effective and accessible way, which otherwise would be costly and unsustainable [12]. In addition, eHealth interventions have shown positive impacts on knowledge, social support, and clinical and behavioral outcomes [18]. Johnson et al have highlighted the benefits of eHealth interventions on T2D management, such as increased support, self-efficacy, and knowledge; improvements in glycemic levels and self-management behaviors; and efficient use of primary care services [12]. Furthermore, successful eHealth programs focused on DSME provided relevant content, engaging interactive elements, personalized learning experiences, and self-assessment tools for monitoring and feedback [17-20]. However, in spite of the potential benefits eHealth offers for DSME, eHealth interventions have been mostly based on traditional website formats. Such website formats generally lack realistic simulated environments where DSME actually takes place, such as patient community places (eg, grocery stores and restaurants) [7,21].

### Virtual Environments and Diabetes Self-Management Education and Support

Virtual environments offer an effective way to provide patients with realistic settings for the acquisition and application of knowledge in community settings where daily T2D self-management takes place, while addressing barriers such as transportation, cost, time, and scheduling issues [22]. In addition, virtual environments have started to show a potential to improve diabetes self-efficacy and social support, while being highly feasible and usable [12]. Second Life (Linden Lab), a highly popular virtual world, has been shown to be an effective tool that can lead to “significant learning gains” [23]. Second Life allows users to socialize and behave in a similar way as they would naturally do in normal settings through virtual human representations known as avatars [24]. Furthermore, virtual environments, such as Second Life, offer the potential for users to perform behaviors within realistic scenarios by providing them with presence, immersion, and social interaction, while facilitating communication between patients, educators, and providers [12,24]. While virtual environments have been used to deliver health information, education, social support, and social networking, most Second Life–based health sites to date have focused on disseminating information and offering support groups [24].

Self-management diabetes interventions based on virtual environments enable diabetes education, the development of new skills, and the exchange of peer support in synchronous and asynchronous ways [7]. The Second Life Impacts Diabetes Education & Self-Management (SLIDES) virtual community was among the first interventions aimed at providing DSME and support using Second Life [24]. The results of SLIDES showed improvements in diabetes self-efficacy, social support, and foot care, as well as trends toward improvements in diet, weight loss, and clinical outcomes, while being highly feasible and usable [12]. The development of the SLIDES platform, as well as its preliminary effects, is described elsewhere [12,24]. Virtual environments, such as SLIDES, are innovative ways to provide accessible DSME and ongoing self-management support. A key characteristic of these environments is the potential for participants to develop real-world skills via simulation and rehearsal within the virtual environment that can be transferable and thus affect behaviors in the real world [12].

Another significant characteristic of virtual environments is the facilitation of social support among participants [12,24]. Social support is generally described as “an exchange of resources between at least two persons aimed at increasing the wellbeing of the receiver” [25-27]. Social support is recognized as a key component of diabetes self-management, in addition to adequate skills and behavioral development [22,28,29]. Studies have shown that social support is commonly provided through social interactions to achieve health outcomes [30,31]. Moreover, research suggests that people with T2D can benefit from frequent and sustained social interactions among peers and providers by obtaining education and support [28,32-34]. In addition, T2D interventions that are based on virtual environments can provide realistic, personalized, and ongoing interaction and support that assist participants in health care decision making [7,12,34-36]. SLIDES showed that virtual environment–mediated interactions resemble physical ones; therefore, patients with T2D are presented with the possibility of greatly improving their access to social support [12,34]. However, the social networks highlighting the patterns of interactions within T2D-specific virtual communities, such as SLIDES, have not been studied. While the prominent effects of social relationships on health decisions and related behavior changes have been established [37,38], little is known about social interactions and the exchange of support in disease-specific virtual environments.

### Social Network Analysis and Online Health Communities

The study of social networks provides researchers with a unique opportunity to get an in-depth view and a better understanding of the structure of online communities [38,39]. Social network research has shown that social connections (ie, peers, family members, etc) disseminate health information, provide social support, and influence health behaviors [38,39]. Social network analysis (SNA) has been used to study the ways in which social connections can influence individuals’ attitudes, believes, and behaviors. Such network influences can be caused by the network environment, the position an individual occupies in the network, or structural or network-level properties [38,39]. For example, being central in a social network determines a high importance for information dissemination. Similarly, individuals located on a network’s periphery, known as peripheral individuals, can act as bridges connecting otherwise disconnected groups, thus enabling collective actions. Peripheral individuals are characterized by having one or few connections on the outside of a network and thus participating infrequently. Moreover, peripheral individuals are usually free from social norms and constraints, and thus, innovation can occur [38,39]. Furthermore, network structural properties, such as clustering, can help to identify highly connected groups of individuals, where behavior change can be accelerated. Lastly, densely connected networks have been shown to generate faster diffusion and increased coordinated action [38,39].

SNA is increasingly becoming useful to the study of online health communities owing to the exponential growth in the use of electronic communications [40]. The massive amounts of social interactions taking place within online communities today are providing researchers with valuable network data. Research has focused on the analysis of online social interactions from both general purpose social media platforms (eg, Twitter and YouTube) and health care–specific platforms (eg, American Diabetes Association online community) [41-44]. Often, qualitative analysis and computational text analysis are used to analyze social media interactions [41-43]. Studies have shown that SNA provides insights into social influence, information dissemination, and behavioral diffusion [39,40,45,46]. On one hand, communication structure (who communicates with whom) is key for the study of peer influence on health behaviors [40]. On the other hand, analyses of the structures of online peer-to-peer communications provide valuable insights into opinion leaders [40,45,47]. Both approaches have the potential to help researchers model effective network data–based interventions [40]. Similarly, social support exchange patterns within disease-specific virtual communities, such as SLIDES, can be studied using a SNA approach, which would allow the visualization and description of communication structures, peer influences, and behavioral diffusion, as well as the impact on health outcomes, such as blood glucose levels, for patients with diabetes [45-50]. However, despite the benefits SNA offers, to our knowledge, social interactions occurring within virtual environments have not been studied using this approach. In this study, a secondary data analysis of SLIDES social interactions through the SNA lens was carried out to examine social support exchange patterns between participants and providers [12,24,34].

### Research Aims

The overall goal of our study was to examine social support exchanges from a T2D self-management education and support intervention (SLIDES) that was delivered via a virtual environment. The specific aims of our study were as follows: (1) to examine patterns of social interaction and support of the SLIDES intervention by creating network structures for different types of social supports and assessing these support networks using quantitative network measures; (2) to explore the associations between social support network structures by correlating them with each other using the quadratic assignment procedure (QAP); and (3) to provide insights into the exchange of social support within a disease-specific virtual environment.

## Methods

### SNA Methodology

#### Social Network Data

SLIDES social interaction data were used for our study [34]. SLIDES included a total sample of 24 individuals, with 20 participants and 4 providers (including diabetes educators and moderators). Detailed participant demographics are described elsewhere [12]. SLIDES facilitated virtual interactions among participants with T2D and providers in the following two types of sessions: education and support. Education sessions were held twice a week, and support sessions were held weekly. SLIDES social interactions consisted mostly of synchronous naturalistic conversations that took place throughout different locations within the virtual environment (eg, bookstore, restaurant, and classroom) [12,24]. These conversations enabled the exchange of social support among participants and between participants and providers, and were continuously recorded and transcribed [12,24]. These transcriptions provided the data set from which network data were derived for our analysis. Detailed information on the SLIDES study site, theoretical framework, sample, measures, and outcomes have been published elsewhere [12,24]. Our analysis focused on interactions where social support was exchanged among participants and between participants and providers during a 6-month study enrollment period [34]. Study participants could log into SLIDES and participate as much or as little as they wanted and engage in synchronous conversations. Social support was defined as “personal informal advice and knowledge that help individuals initiate and sustain T2D self-management behaviors, thus increasing adherence” [22,25,27,30,34]. Social support types included emotional, instrumental, informational, and appraisal [22,25-27,29,34]. SLIDES social interactions, which were previously characterized by the aforementioned types of social support [34,51], were used to create network structures in order to analyze social support exchange patterns at the group level (ie, participants/providers who interacted in a conversation by either listening or engaging directly, where a certain type of support was exchanged, were all linked together for that particular conversation). Thus, the unit of analysis included the tie among participants and between participants and providers who interacted via synchronous conversations, as well as the types of social support exchanged in each transcribed conversation as previously characterized [34,51].

#### Network Structures and Measures

Network structures were created for each type of social support by representing participants and providers as nodes and representing interactions where social support was exchanged as edges (interconnections between nodes). For each type of social support network, all edges indicating who participated in a conversation were included (ie, who interacted with whom during a virtual conversation in which social support was exchanged). Quantitative network measures were used to assess network structures across all types of social support. Network measures explain structural differences (eg, density and cohesion), as well as node importance within a network (eg, centrality) [38,39]. The following network measures were used: *average degree* (average number of connections of all nodes; a higher average degree number means that members of a network interacted with a higher number of members via synchronous conversations, either on a one-to-one basis or at a group level); *graph density* (proportion of connections relative to the total number of possible connections; ranging from 0 to 1; a higher graph density means that members of a network most likely engaged in conversations involving a higher number of members, ie, larger groups); *average path length* (average distance between all node dyads; the distance of a dyad is 1, which means a direct interaction between two members of the network; a higher average path length is associated with a higher distance or number of steps required for two network members to interact with each other, resulting in a less efficient network); *average clustering coefficient* (average measure of the interconnectivity of the node neighborhood; ranging from 0 to 1; a higher average clustering coefficient means that node neighborhoods are more interconnected, indicating conversations among a larger number of members for larger node neighborhoods); and *modularity* (the level of development of subcommunities within a network; ranging from −1 to 1; higher modularity values indicate higher levels of subcommunity development within a network) [38,39].

#### Network Statistical Analysis

Once network structures were created, we correlated them with each other to explore associations between social support network structures. The QAP was used to test network correlations. QAP is a nonparametric method based on permutations that allows testing structural similarities (correlations) between social network structures [52]. We used Gephi version 0.9.2 and UCINET version 6.685 (Analytic Technologies) to create network structures and to calculate network measures, as well as to perform correlation analysis [53,54].

## Results

### Network Structures

**Figure 1:**
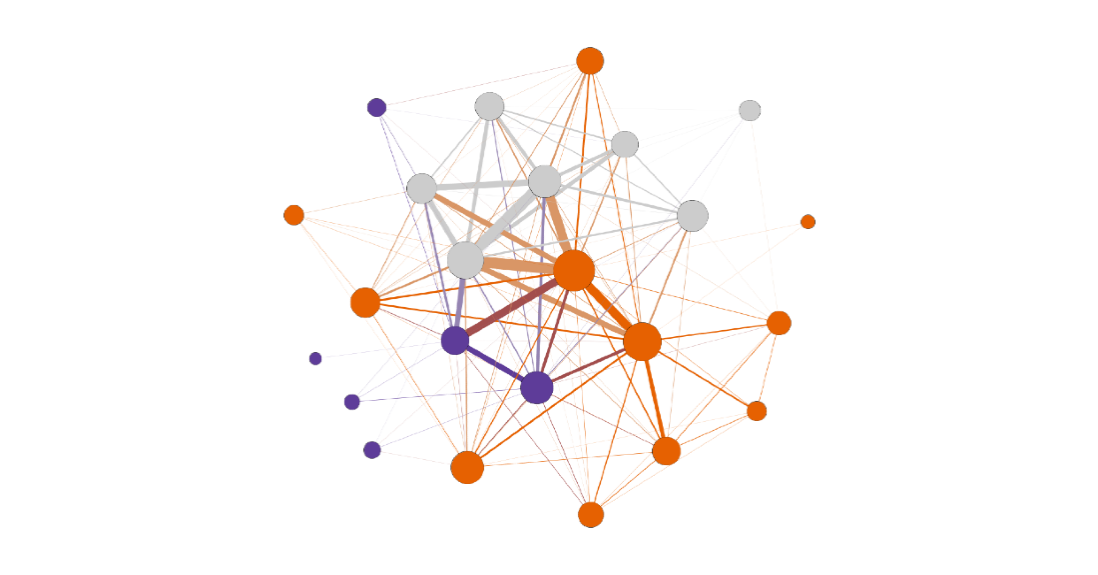
Network structure of social interactions where all types of social supports were exchanged. Node size indicates degree and node color indicates the existence of three subcommunities or groups, with one larger subcommunity shown in orange and two smaller subcommunities shown in purple and grey. Further, edge thickness represents the frequency of interactions when members communicated more often.

**Figure 2:**
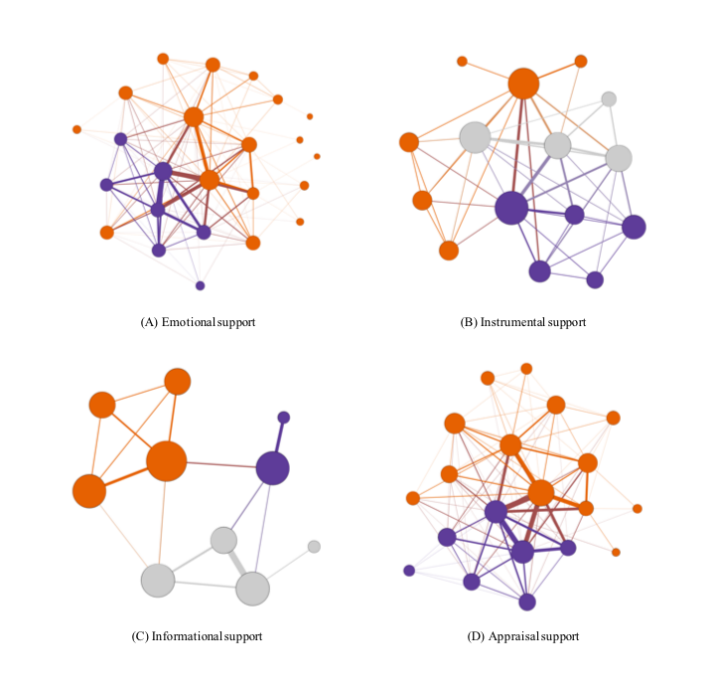
Network structures of Second Life Impacts Diabetes Education & Self-Management (SLIDES) social support interactions by the type of support. Node size indicates degree and node color indicates the existence of subcommunities, where larger subcommunities are shown in orange and smaller subcommunities are shown in purple and grey.

Figure 1 shows a network structure depicting all SLIDES social interactions where all types of social support were exchanged among participants and between participants and providers. Network structures for each type of social support exchanged by SLIDES participants are shown in Figure 2.

In addition, Table 1 summarizes the network measures for each social support network. As seen in Figure 2, the emotional and appraisal support networks were the most populous, with the former comprising 24 nodes and 1219 edges and the latter comprising 20 nodes and 737 edges. Moreover, the emotional and appraisal support networks had the highest average degrees (9.08 and 9.5, respectively) compared with the instrumental and informational support networks (6.0 and 3.2, respectively). This indicates that each member of these support networks interacted on average with nine other members via synchronous conversations, either on a one-to-one basis or at a group level, thus making them the most active networks. Additionally, assessment of degree at a node level showed that all support networks were somewhat centralized around a few nodes, suggesting that some members were more popular. Furthermore, the appraisal (0.5) and instrumental (0.43) support networks were the densest, suggesting that members of these networks most likely engaged in conversations involving a higher number of members (ie, larger groups), where some participants directly exchanged appraisal and/or instrumental support, while other members of the group had a latent exposure to this support.

**Table 1:** Summary of social network metrics for Second Life Impacts Diabetes Education & Self-Management (SLIDES) social support networks.

Social support network	Average degree	Graph density	Average path length	Clustering coefficient	Modularity
Emotional	9.08	0.39	1.74	0.73	0.11
Instrumental	6.0	0.43	1.62	0.76	0.12
Informational	3.2	0.35	1.98	0.57	0.46
Appraisal	9.5	0.5	1.52	0.72	0.12

Additionally, no substantial differences were observed between all average path length values. However, the appraisal (1.52) and instrumental (1.62) support networks had a slightly lower average path length compared with the emotional (1.74) and informational (1.98) support networks. This indicates that the distance or number of steps needed for members of these networks to interact with each other required on average fewer steps to exchange the supports, thus making these networks more efficient. In terms of network structure and community development, on one hand, the instrumental, emotional, and appraisal support networks had higher average clustering coefficients (76%, 73%, and 72%, respectively) compared with the informational support network (57%). These results indicate high levels of interconnectivity within these support networks. On the other hand, the modularity values of the emotional (0.11), appraisal (0.12), and instrumental (0.12) support networks were lower compared with that of the informational (0.46) support network. This indicates that subcommunities of network members exchanging informational support reached higher levels of development in comparison with subcommunities from all other support networks.

**Figure 3:**
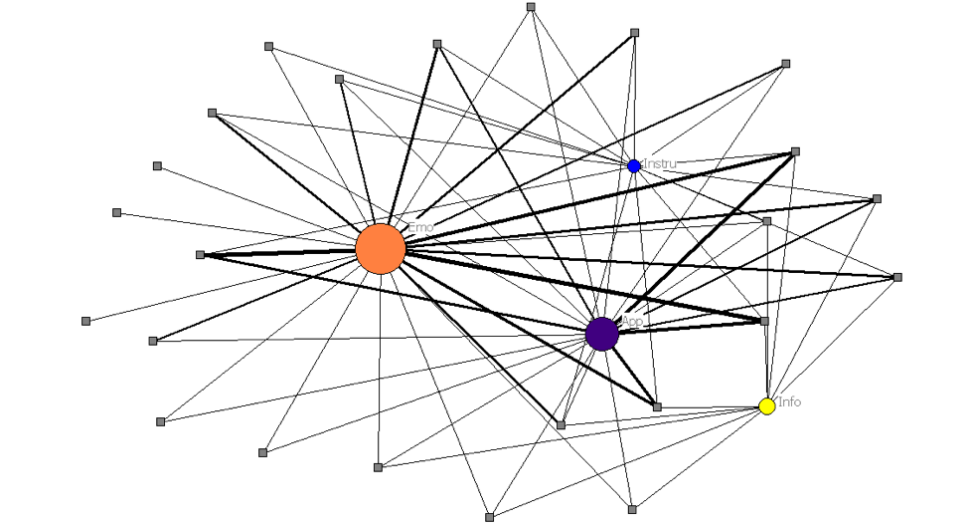
Two-mode network structure of social interactions for all types of support. The shape of the nodes distinguishes two sets of nodes as follows: squares represent participants and providers, and circles represent types of social support. In addition, the color of the circles represents each type of social support (orange, purple, yellow, and blue representing emotional, appraisal, informational, and instrumental support, respectively). Finally, the size of the circles indicates degree, and edge thickness represents the frequency of participants’ interactions within each type of support.

Lastly, Figure 3 illustrates a two-mode network representing the affiliation between participants and providers, and the types of social support exchanged via social interactions. As seen in Figure 3, according to degree, the two-mode network is centralized around emotional and appraisal support, indicating that a higher number of participants and providers participated in interactions where these types of support were exchanged (either directly or indirectly having a latent exposure as previously discussed). Moreover, a subgroup of participants and providers engaged more frequently in interactions where emotional support and appraisal support were exchanged, which are represented by thicker edges.

### Network Statistical Analysis

Table 2 shows network correlation scores obtained by QAP analysis. All social support networks were correlated with one another. QAP correlation scores between the emotional and appraisal, instrumental and appraisal, and instrumental and emotional support networks were much stronger when compared with the correlations between the informational and appraisal, informational and emotional, and instrumental and informational support networks. The stronger correlation scores suggest that considerable similarities exist between the aforementioned social support networks.

**Table 2:** Network correlation test results.

Variable		Appraisal	Emotional	Informational	Instrumental
**Appraisal**					
	Score	1	0.974	0.344	0.833
	*P* value	—^a^	<.001	.004	<.001
**Emotional**					
	Score	0.974	1	0.318	0.818
	*P* value	<.001	—	.003	<.001
**Informational**					
	Score	0.344	0.318	1	0.204
	*P* value	.004	.003	—	.02
**Instrumental**					
	Score	0.833	0.818	0.204	1
	*P* value	<.001	<.001	.02	—

^a^Not applicable.

## Discussion

### Principal Findings

In this study, we used SNA to examine patterns of social interactions and support of SLIDES, an intervention for T2D self-management education and support that was delivered via a virtual environment [12,24]. To the best of our knowledge, this study is among the first to explore the patterns of social interactions of a disease-specific virtual environment. This novel approach provided insights into the exchange of social support within the SLIDES virtual community. Our findings indicate that emotional and appraisal support networks were the largest, most centralized, and most active, indicating that a virtual community with a larger number of members can be more supportive. Moreover, a higher centralization indicated that some network members were more active, which suggests that a virtual community benefits from having active members, such as educators and moderators, because they can help engage the community. This is important for the design of interventions based on virtual environments. For example, interventions could recruit diabetes moderators or leaders to act as peer influencers or change agents. Moreover, appraisal and instrumental support networks are more connected than emotional and informational support networks. This suggests that more members are likely to engage in larger group synchronous conversations, thus indicating that well-connected networks can facilitate the exchange of appraisal and instrumental support within virtual communities. This finding could be leveraged when designing interventions that facilitate the exchange of appraisal and/or instrumental support.

An analysis of the structures of the support networks revealed higher levels of interconnectivity within the instrumental, emotional, and appraisal support networks, as indicated by their higher average clustering coefficients. Clustering can accelerate information and behavior spread [38,39], thus suggesting that interventions based on virtual environments can leverage this characteristic to accelerate the exchange of social support. Despite high degrees of clustering, instrumental, emotional, and appraisal support networks had low modularity values, indicating low levels of subcommunity development. In contrast, the informational support network showed a higher level of subcommunity development. From an intervention’s perspective, subcommunities or groups within informational support networks can be leveraged to spread resources and behaviors, in addition to providing informational support. Studies have shown that groups have norms and exert social pressure, enabling behavior change, as well as more opportunities to access information, resources, and support [39].

Our findings also show that a higher number of participants and providers participated in interactions where emotional support and appraisal support were exchanged, and they did so more frequently. These findings diverge from a previous analysis by Lewinski et al, where informational support and emotional support were the most commonly exchanged types of support among participants and between participants and providers, and appraisal support exchange was lower [34]. Their analysis focused on support exchanges at a dyadic level in order to characterize interactions. In contrast, our analysis focused on support exchanges at a group level, as previously indicated. In other words, a dyadic analysis for two participants who interact in a group conversation would identify the frequency of support exchanged between those two participants. On the other hand, our network approach to this same scenario would take into account the connections between all participants who engaged in the conversation, including those who actively engaged one another to exchange support, as well as the other participants who engaged passively and had a latent exposure. Taking this into account, we hypothesize that a higher and more frequent engagement in interactions where emotional and appraisal support were exchanged was caused by the role providers, specifically diabetes educators, played assisting in the self-management of diabetes.

Lastly, network correlations showed that all social support networks were correlated with one another. Specifically, stronger correlation scores for emotional and appraisal, instrumental and appraisal, and instrumental and emotional support networks indicate that considerable similarities exist between these networks. These results suggest that SLIDES participants who exchanged emotional support were likely to exchange appraisal or instrumental support. Likewise, participants who exchanged appraisal support were likely to exchange instrumental support. From an intervention’s perspective, educators and moderators from virtual communities can leverage interactions where a certain type of support is exchanged in order to maximize the provision of advice and support among members of such communities. For example, by promoting interactions between members where emotional support is exchanged, further discussion and opportunities could be created that would most likely prompt exchange of appraisal or instrumental support [34,55,56]. As a result, a higher number of supportive relationships would be fostered among participants and providers, increasing the effectiveness of support networks and thus substantiating the value of virtual communities for diabetes self-management and other health goals.

### Limitations

There are several limitations in this study. The small sample size of the SLIDES study (N=24) created a small virtual community, which consequently resulted in a small community. The social dynamics resulting from a small community might differ from larger ones, which suggests that our findings should be interpreted with caution. The creation of social networks from interactions, where some type of social support was exchanged, was considered at a group conversational level and not at a dyadic level. This resulted in group identification of social support interactions, meaning that a type of social support was assigned to all group participants interacting in a conversation where social support occurred during a particular conversation. Future studies could improve network creation by analyzing participants’ interactions at a dyadic level so that social support exchanges describe social ties at a dyadic level, thus providing more accurate social support dynamics. Despite these limitations, we consider these findings valuable because of the insights provided into social support exchanges within disease-specific virtual environments.

### Conclusions

This study described the utility of SNA to examine social support in a DSME virtual environment. Our findings have revealed structural differences between support networks, as well as key network characteristics of supportive interactions facilitated by the virtual community, with emotional and appraisal networks being large, centralized, and most active, thus emphasizing the value of virtual environments as sources of these two support types for T2D patients. In addition, support networks have highlighted the benefits central members, such as educators and moderators, can contribute by facilitating community engagement. Specifically, educators and moderators from the SLIDES intervention have facilitated community engagement by leading weekly synchronous group meetings that include educational sessions, focusing on core American Diabetes Association/American Association of Diabetes Education self-management curriculum, as well as support sessions [12].

Furthermore, our appraisal and instrumental support networks suggest that members of virtual communities are more likely to engage in larger group interactions where these types of support can be exchanged, with the caveat that some members can engage one another to actively exchange support, while the other members engage passively and have a latent exposure to support exchange. Lastly, our network correlation analysis has shown that participants who exchange emotional support are likely to exchange appraisal or instrumental support, and participants who exchange appraisal support are likely to exchange instrumental support. These associations suggest that interactions, where a certain type of support is exchanged, could be leveraged to maximize the provision of advice and support among network members, thus increasing the effectiveness of support networks enabled by virtual communities.

Network data can provide valuable insights into the design of novel and effective digital health interventions given the unique opportunity disease-specific virtual environments have facilitating realistic environments that are effective and sustainable, where social interactions can be leveraged to achieve diverse health goals.

## Abbreviations

DSME: diabetes self-management education
QAP: quadratic assignment procedure
SLIDES: Second Life Impacts Diabetes Education & Self-Management
SNA: social network analysis
T2D: type 2 diabetes
